# Pure uniportal video-assisted thoracic surgery for treating thoracic tuberculous spondylitis: an initial case series of seven patients

**DOI:** 10.1186/s13018-023-04113-9

**Published:** 2023-08-29

**Authors:** Xin Xiu, Yungang Chen, Yonghua Ding, Qiang Zhang, Deqiang Chen

**Affiliations:** 1 Shandong University of Traditional Chinese Medicine, Jinan City, Shandong Province China; 2 Affiliated Hospital of Shandong University of Traditional Chinese Medicine (Shandong Provincial Hospital of Traditional Chinese Medicine), Jinan City, Shandong Province China; 3 Public Health Clinical Center Affiliated to Shandong University, Jinan City, Shandong Province China

**Keywords:** Pure uniportal, Video-assisted thoracic surgery, Tuberculosis, Thoracic vertebra

## Abstract

**Background:**

The development of thoracic surgical techniques has provided a new avenue for treating thoracic tuberculosis. Moreover, microscopic treatment of spinal tuberculosis has attracted increasing attention, as it affords good visual access and reduces trauma. Traditional thoracoscopic treatment of spinal tuberculosis usually requires 2–3 passages, accompanied by a corresponding number of incisions. With a large number of conventional thoracoscopic surgeries performed, improved resolution of the microscopic field of view, effective hemostasis of the peripheral vessels using the ultrasonic knife, and many reports in the literature, thoracic tuberculosis can now be treated microscopically by creating a single channel. The aim of this study was to explore the feasibility and surgical technique for thoracic tuberculous spondylitis treatment via debridement and bone graft fusion surgery employing pure uniportal video-assisted thoracic surgery (VATS), combined with posterior internal fixation.

**Methods:**

Seven patients with relatively complete documentation were included in this study. All patients underwent lesion removal and bone graft reconstruction via uniportal VATS with posterior internal fixation. The mean patient age was 39.6 years. Surgical duration, blood loss volume, postoperative recovery time, and thoracic kyphosis angle were recorded.

**Results:**

The surgeries were successful with no severe postoperative complications. All patients were followed-up, and no recurrence of tuberculosis was observed. Imaging data, including computed tomography scans, confirmed the complete removal of the lesions. Additionally, bone fusion at the graft site was successful, no loss of the thoracic kyphosis angle was noted postoperatively, and the thoracic kyphosis angle improved.

**Conclusions:**

Pure uniportal VATS yields satisfactory results and inflicts less trauma than previous surgical techniques. This technique also offers a reference value for treating thoracic tuberculous spondylitis.

**Supplementary Information:**

The online version contains supplementary material available at 10.1186/s13018-023-04113-9 .

## Background

Spinal tuberculosis, the most severe form of osteoarticular tuberculosis, is a major cause of paraplegia in developing countries [1]. The relatively fragile thoracic spine is frequently affected by this condition with the primary clinical manifestations including spinal deformities and localized back pain [2]. Although conservative treatment can effectively relieve pain, adequate management of thoracic kyphosis and paravertebral abscesses is difficult. Additionally, this disease often affects multiple vertebrae; destruction of multi-segment vertebrae increases the risk of paraplegia and thoracic kyphosis by approximately 3–5% [2, 3]. Therefore, surgical intervention is imperative for patients unresponsive to antituberculosis drugs or who have already developed thoracic kyphosis and paraplegia.

Conventional surgical strategies for thoracic tuberculosis include the anterior, posterior, and combined anterior–posterior approaches [4, 5]. In 1960, Hodgson et al. reported a method involving anterior decompression and autologous bone grafting to treat thoracic tuberculosis [6]. Subsequently, anterior surgery has been considered the gold standard method for the surgical management of spinal tuberculosis [7, 8] and is widely applied in clinical practice owing to its capacity for complete exposure of the lesion and thorough debridement. However, anterior surgery has inherent limitations, including failed correction due to the low fixation strength of the internal fixator, severe trauma and vascular damage due to complex thoracic anatomical structures, and surgical intolerance in patients with poor pulmonary function due to significant pulmonary impact [9–12]. With the development of thoracic surgical techniques, video-assisted thoracic surgery (VATS) has been used to treat spinal diseases.

Compared with traditional open surgery, VATS offers notable advantages including fewer associated injuries, shorter postoperative recovery time, lower postoperative pain levels, and relatively smaller surgical scars [13, 14]. Based on the original VATS technique, the current study used pure uniportal VATS for debridement and bone grafting fusion surgery to treat patients with thoracic tuberculosis, achieving satisfactory therapeutic outcomes.

## Methods

### Patient selection

Seven representative cases from a larger patient group, including two men and five women, were selected for this surgical treatment. The mean patient age was 39 years (range: 29–50 years). Lesions were present in a single segment in two cases and spanned across multiple segments in the remaining five cases.

The inclusion criteria were: ① definitive clinical, radiological, or pathological diagnosis of thoracic tuberculosis; ② persistent, severe back or radicular pain following conservative treatment; ③ neurological dysfunction due to an abscess or intervertebral disk tissue compression.

The exclusion criteria were: ① a combination of cervical or lumbar diseases; ② severe cardiopulmonary dysfunction; ③ inability to tolerate single-lung ventilation or severe pleural thickening and adhesion preventing thoracoscopy; ④ concurrent malignant tumors or other malignancies. Each patient received a comprehensive explanation of the surgical risks, benefits, and drawbacks before surgery and provided signed informed consent.

### Preoperative preparation

Antituberculosis drugs (rifampicin, isoniazid, pyrazinamide, and ethambutol) were administered regularly and in combination for at least 4 weeks before surgery. The complete erythrocyte sedimentation rate (ESR), C-reactive protein (CRP) level, routine blood tests, and tuberculosis antibody laboratory tests were performed. The preoperative mean white blood cell count was 5.14 × 10^9^/L (range: 3.22–9.72 × 10^9^/L), mean ESR was 51.4 mm/h (range: 6–108 mm/h), and mean CRP concentration was 26 mg/L (range: 8.5–62.4 mg/L). Radiological examinations, including radiography, CT, and MRI, were performed. The average thoracic kyphosis angle was 17° (range: 9°–25°), obtained by measuring the superior endplate of the upper vertebra and the inferior endplate of the lower vertebra. Surgery was performed after a final evaluation of the surgical risks, ensuring no notable contraindications.

Considering spinal stability, bone graft efficacy, and ease of implant removal post-recovery, we opted for combined posterior internal fixation surgery. Before the surgery, we discussed with each patient whether to perform posterior internal fixation surgery and pure uniportal thoracoscopy simultaneously; the decision was made based on their preferences and physical state. Three patients underwent simultaneous surgery, whereas four patients underwent thoracoscopic surgery after internal fixation surgery.

### Surgical process

**Fig. 1:**
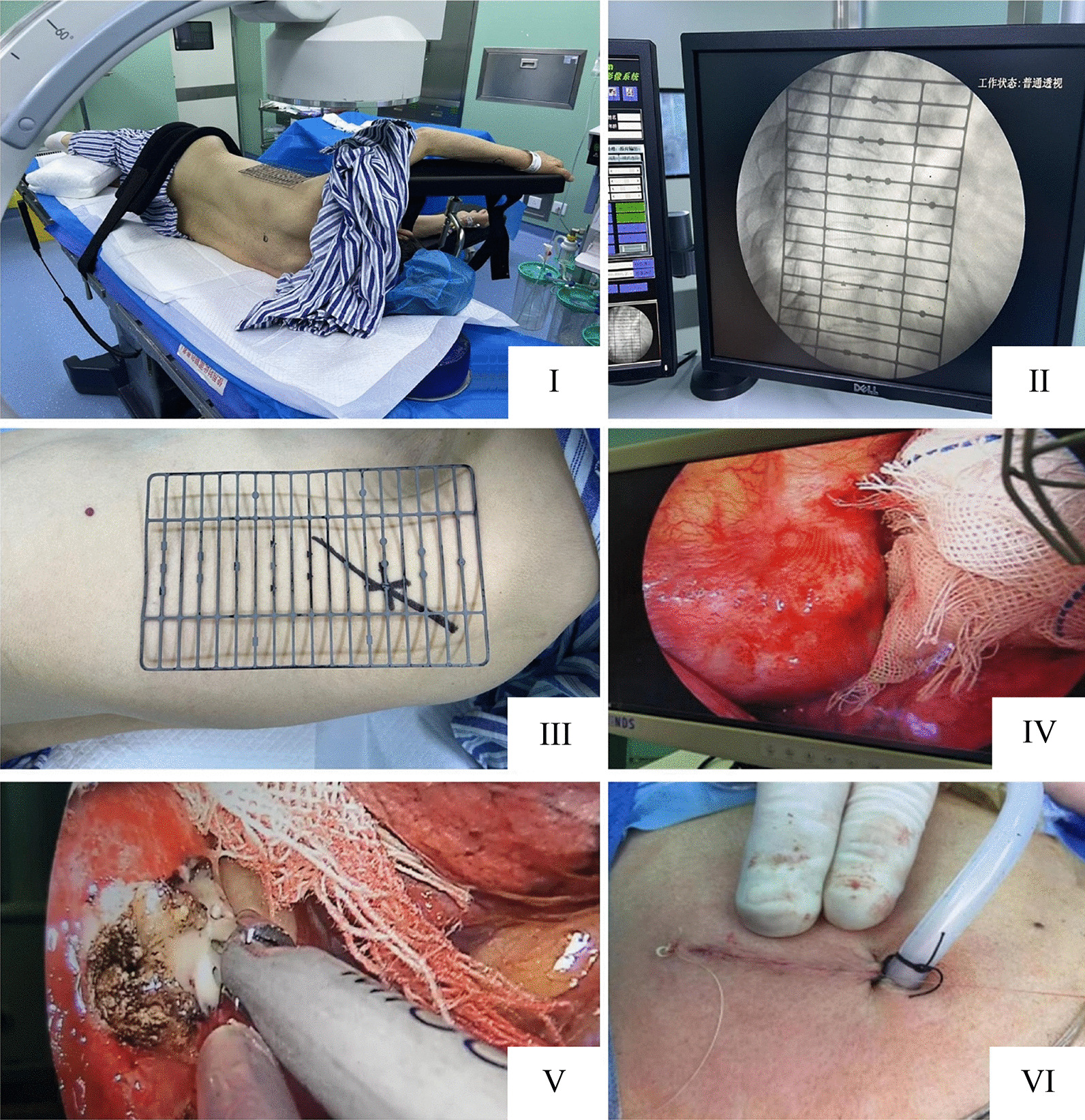
Key to the surgery is preoperative positioning. (I) The required standard lateral position before the operation. (II) No ghost image was present under fluoroscopy. (III) Markings on the body surface. (IV) The lesion under the thoracoscope. (V) The aspirator, instruments, and lens passing through a single passage simultaneously for lesion removal. (VI) The placement of the postoperative drainage tube and suturing of the incision

Surgery was performed under general anesthesia with the patient in the lateral position and with their upper limbs abducted and affixed to the arm frame **(**Fig. 1**)**. For ipsilateral lung collapse and single-lung ventilation, a double-lumen endotracheal tube was inserted, and all hemodynamic and respiratory system parameters were closely monitored. Based on the radiological examination results and considering the abscess volume and extent of bone destruction, the patient was placed in the left or right lateral position. Cloth strips were applied over critical areas, and preparations were made for standard posterolateral thoracotomy.

Using fluoroscopy, the precise locations of the affected vertebrae and intervertebral space were identified to determine the optimal position of the surgical pathway. To select the incision site, a line parallel to the intercostal space was marked at the projected position of the affected intervertebral space on the body surface. The starting point of the line was perpendicular to the posterior edge of the affected vertebrae (close to the posterior axillary line) and extended laterally toward the chest. The overall length ranged from 5 to 7 cm. This strategy was used to establish an incision and obtain a good grafting angle.

Following completion of the pathway incision, the soft tissue was further treated to establish a pathway. Similar to a thoracotomy, the thoracic cavity was opened for examination. In instances of mild adhesion between the lung and chest cavity, blunt separation was performed, and the lung tissue was freed successfully. An ultrasonic scalpel was used to access the lesion via the pathway, and a periosteal elevator was used to bluntly separate the lung tissue from the chest wall. Segmental vessels obstructing the view or pathway establishment were severed using an ultrasonic scalpel (Additional file 1), the anterior vertebral fascia at the affected vertebral abscess location was incised using an ultrasonic scalpel, and pus was removed using an aspirator. Curettes, nucleus pulposus forceps, and a Kerrison rongeur were used to eliminate dead bone, damaged intervertebral disks, and granulation tissues to establish the graft bed. After flushing the lesion, the allogeneic bone was shaped to fit the size of the vertebral defect, implanted into the vertebral space, and tapped to fit tightly, leaving the prevertebral fascia open. The thoracic cavity was thoroughly flushed to achieve hemostasis. A thoracic drainage tube was positioned next to the affected vertebrae to ensure complete drainage, and the incision was sutured.

## Results

**Fig. 2:**
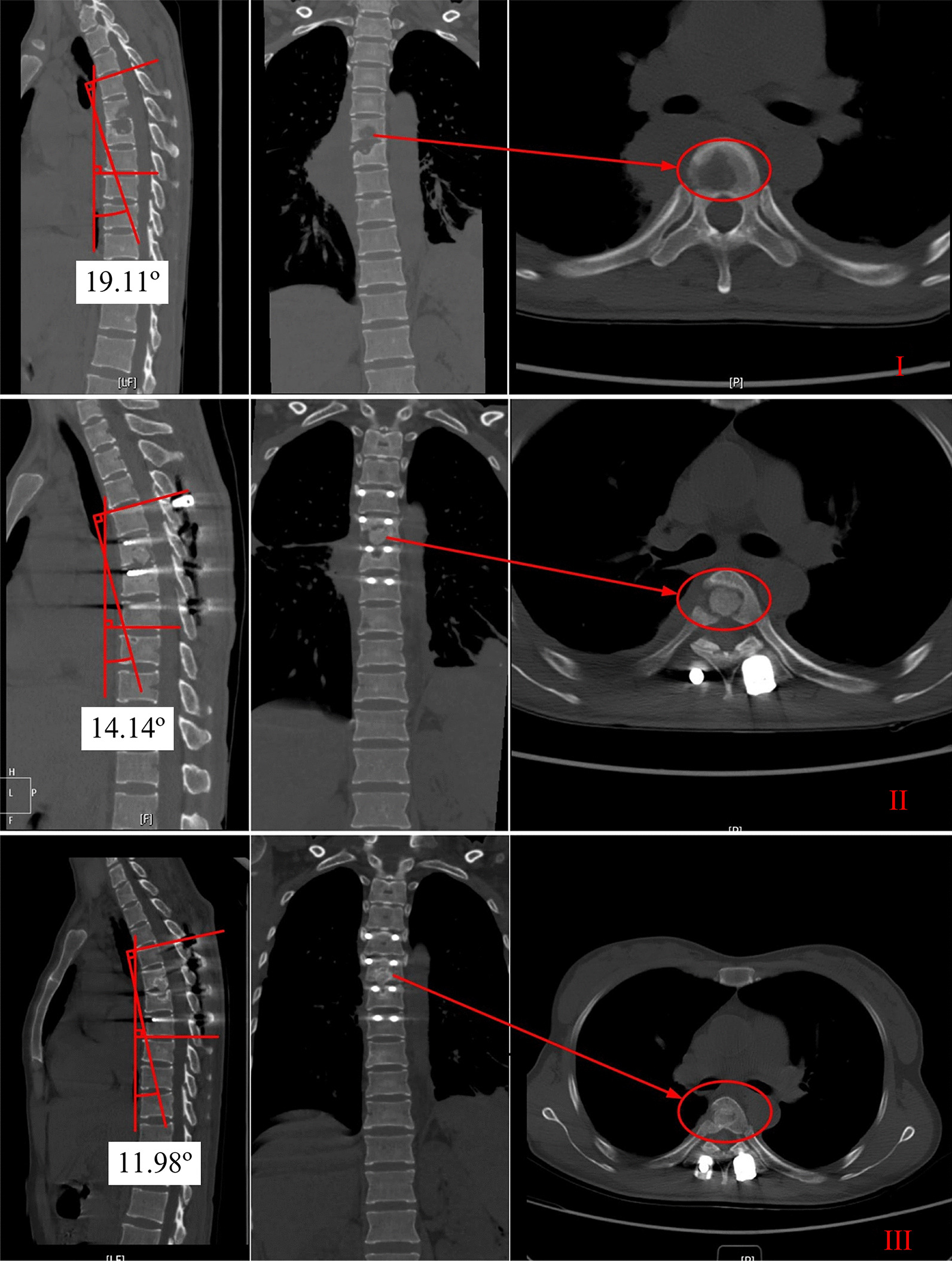
Radiological examinations of a 37-year-old female patient. (I) The CT scan at admission showed severe bone destruction and bone defect. (II) The first postoperative CT showed that the lesion had been completely cleared. (III) The 6-month post-surgery follow-up showed completed bone fusion with no loss of the thoracic angle in the patient

The surgery lasted for an average of 188 min (range: 140–220 min). This wide variability was primarily due to the different numbers of affected vertebral segments involved in each surgery. Moreover, with an increasing number of surgeries performed, the operation time correspondingly decreased. The average blood loss volume was 300 mL (range: 100–600 mL) and increased with operation time. Following surgery, all patients underwent antituberculosis chemotherapy, typically comprising a four-drug regimen (rifampicin, isoniazid, ethambutol, and pyrazinamide). During follow-up, the latest thoracic computed tomography (CT) scans and three-dimensional (3D) reconstructions for each patient were retained, enabling a comparison of the thoracic kyphosis angles preoperatively, postoperatively, and at follow-up. The mean angle at the final follow-up was 10° (range, 5°–14°), with no significant loss in angle (Table 1). Bone fusion was achieved in all patients, with no incidence of implant loosening or neurological or vascular complications. The mean postoperative hospital stay was 21 days (range: 11–40 days; Fig. 2**).** This considerable variation was primarily due to superficial wound infections in two patients, both of whom were successfully treated after debridement.

**Table 1:** Patient information

Case	Age	Sex	Segment (s)	Simultaneous surgery	Cobb° (preoperation)	Cobb° (postoperation)	Cobb° (final)	Relapse
1	31	M	T7–9	Yes	17.25	10.62	6.76	No
2	37	F	T6	Yes	19.11	14.14	11.98	No
3	50	M	T6–7	Posterior internal fixation on Aug 2, 2022, pure uniportal VATS on Sep 8, 2022	25.59	15.85	13.31	No
4	29	F	T10–11	Posterior internal fixation on Feb 28, 2022, pure uniportal VATS on Mar 11, 2022	12.58	11.53	9.18	No
5	42	F	T8–9	Posterior internal fixation on Jun 10, 2022, pure uniportal VATS on Jun 23, 2022	9.26	5.55	3.96	No
6	50	F	T10	Posterior internal fixation on Jul 21, 2022, pure uniportal VATS on Sep 2, 2022	16.76	11.05	6.76	No
7	38	F	T7–8	Yes	19.91	14.43	10.88	No

No thoracic angle was lost after the surgery

## Discussion

The starting point of this study was the search for a less invasive surgical approach for the treatment of thoracic spine tuberculosis, and a bold attempt after we had accumulated considerable experience with open and conventional thoracoscopic surgery. During the course of our research, we found that the use of ultrasonic knife for ligation of thoracic segmental blood vessels has been very helpful, and along with the improvement of lens resolution, a series of surgical operations such as separation of tissues, hemostasis, cleanup of lesions, and implantation of bone, which used to be performed by two or even more channels, can be accomplished by a single channel. Of course, we have successfully treated a large number of patients with satisfactory results using this procedure; however, the number of complete cases is relatively small due to the short duration of the procedure and the personal factors of the patients, many of whom were not able to maintain a complete follow-up.

The use of thoracoscopy for the treatment of tuberculosis was first reported by Huang et al. in 2000 [15]. This method provides a panoramic view of the thoracic structure from T2 to T12, providing a clearer and more extensive surgical field than traditional thoracotomy does. Previously, VATS typically involved three incisions: observation port, auxiliary port, and main operation port. However, recent advancements in VATS have enabled two-incision operations, including a 1.5 cm observation port and a 5–7 cm main operation port [16–18]. This procedure was further improved by eliminating the observation port and inserting the thoracoscope and instruments through a single main operating port into the thoracic cavity. The pure uniportal VATS technique represents a bold innovation in surgical practice. Compared with traditional VATS, it results in few postoperative scars and reduces the complications associated with multiple channels. McAfee et al. [19] summarized the outcomes of 100 multicenter clinical studies on endoscopic anterior thoracolumbar spinal reconstructive surgery and reported 16 complications (16%, with one instance of cannula damage to the diaphragm leading to perforation). This underscores the necessity of conducting operations under direct vision. The pure uniportal thoracoscopy used in our study, achieved the same effect as that associated with traditional thoracoscopic surgery requiring two incisions while causing no cannula damage to the diaphragm.

There were several key findings from our surgical experience. Typically, traditional VATS is performed through a combination of an observation channel positioned on the posterior axillary line and an operation channel positioned on the anterior axillary line. In contrast, pure uniportal VATS involves the establishment of a single operating pathway along the entire posterior axillary line. To avoid spinal cord damage, this position must be strictly perpendicular to the surface projection of the lesion and must not exceed the posterior edge of the vertebra. This necessitates careful positioning of the patient in the standard lateral position (under fluoroscopy, the superior endplate of the surgical vertebra aligns with the tangential line and the spinous process is in the center of the vertebra). We encountered a case in which the incision was not perpendicular to the intercostal space of the affected vertebra, and the angle for clearance at the affected vertebra and bone grafting was unsatisfactory. As a result, we refluoroscoped and accurately identified the position before completing the surgery. During the procedure, the surgeon’s instruments should intersect with the assistant’s thoracoscope and coordinate at appropriate times to prevent interference with the operation. Therefore, careful management of intrathoracic adhesions is crucial. Although previous studies have indicated that patients with thoracic tuberculosis often exhibit pleural adhesions and that thoracoscopic surgery should be avoided to prevent substantial bleeding [20], based on our clinical observations, as long as the aorta and main vein are well-protected, blunt dissection can be used for separation when the lungs and pleura are mildly adhered. However, for heavier adhesions, electrocoagulation, a flexible detacher, and, potentially, an ultrasonic scalpel can be used. Typically, the lung tissue is released smoothly. We recommend that initial pure uniportal VATS be performed with the assistance of a thoracic surgeon skilled in managing the thoracoscope to ensure a good visual field. As this surgery retains only one working channel, the proficiency of the thoracoscope holder is crucial. The surgeon must develop proficient VATS surgical skills and the ability to identify tissues under a thoracoscope through systematic clinical training to achieve the expected therapeutic effects. Careful severing and hemostasis of segmental vessels are critical. Owing to long-term inflammation in the tuberculous lesion area, the fragility of the segmental vessels in the tuberculous lesion area of the vertebra increases, which can lead to uncontrollable bleeding during surgery. The primary causes of bleeding include vascular injury, unreliable ligation, hemostasis, and extensive bleeding from surgical wounds. Based on our experience, an ultrasonic scalpel can control most cases of segmental vessel bleeding; however, it must be employed away from the spinal cord. Moreover, ultrasonic scalpels cannot control extensive ooze bleeding. In such cases, thoracotomy should be performed for hemostasis. If uncontrollable massive bleeding occurs, immediate conversion to thoracotomy should be performed. Following tuberculosis infection of the spine, the tubercle bacillus consumes the bone tissue of the anterior spinal column and creates a large bone cavity, thus decreasing the stability of the spine (Fig. 2), which is not conducive to patient recovery. Hence, in our surgical strategy, microscopic guidance ensures that the dead bone and granulated tissue from the diseased vertebrae are thoroughly removed. This further supports the preservation of healthy and less healthy vertebral tissues. The subsequent aseptic implantation of the inactivated allograft bone prevents implant-based secondary infections. In the case of simple posterior internal fixation, all stress is distributed to the internal fixation site owing to the lack of support caused by the presence of bone cavities, leading to an increased risk of broken nails and rods. To avoid this, allograft bone must be implanted after the lesion is thoroughly cleaned.

This study has some limitations. For example, it requires a high level of thoracic anatomy knowledge for both the operator and the assistant, and the learning cycle is long. Additionally, cooperation between the assistant and the main surgeon is crucial because there is only a single operation channel, and poor cooperation between the assistant and the main surgeon may lead to poor switching of instruments and poor provision of the field of view. Lastly, this study has a small sample size and lacks a control group. Considering the aforementioned aspects, we intend to conduct additional iterations of this procedure. This will allow us to enhance our experience and gather a more extensive and comprehensive set of patient data.

## Conclusions

In summary, we found that compared with conventional VATS, pure uniportal VATS causes less injury while maintaining surgical efficacy and offers better aesthetic results. Accordingly, this surgical technique is feasible, effective, and worthy of adoption in clinical practice. In the future, we plan to perform more surgeries of this nature and maintain comprehensive long-term follow-up records.

### Supplementary Information

**Additional file 1.** Clearance of lesions, Video files uploaded in mp4 file format.

## Data Availability

The datasets used and/or analyzed during the current study are available from the corresponding author on reasonable request.

## Abbreviations

3D: Three-dimensional
CT: Computed tomography
VATS: Video-assisted thoracic surgery
